# Primary root response to combined drought and heat stress is regulated via salicylic acid metabolism in maize

**DOI:** 10.1186/s12870-022-03805-4

**Published:** 2022-08-30

**Authors:** Xiaoyi Yang, Xinjie Zhu, Jie Wei, Wentao Li, Houmiao Wang, Yang Xu, Zefeng Yang, Chenwu Xu, Pengcheng Li

**Affiliations:** 1 Jiangsu Key Laboratory of Crop Genetics and Physiology/Key Laboratory of Plant Functional Genomics of the Ministry of Education/Jiangsu Key Laboratory of Crop Genomics and Molecular Breeding, Agricultural College of Yangzhou University, Yangzhou, 225009 China; 2 Joint International Research Laboratory of Agriculture and Agri-Product Safety of Ministry of Education of China, Yangzhou University, Yangzhou, 225009 China; 3 Huaiyin Institute of Agricultural Sciences of Xuhuai Region in Jiangsu, Huai’an, 223001 Jiangsu China; 4 Jiangsu Co-Innovation Center for Modern Production Technology of Grain Crops, Yangzhou University, Yangzhou, 225009 China

**Keywords:** Maize, Primary root, Combination stress, Metabolomics, RNA-seq

## Abstract

The primary root is the first organ to perceive the stress signals for abiotic stress. In this study, maize plants subjected to drought, heat and combined stresses displayed a significantly reduced primary root length. Metabolic and transcriptional analyses detected 72 and 5,469 differentially expressed metabolites and genes in response to stress conditions, respectively. The functional annotation of differentially expressed metabolites and genes indicated that primary root development was mediated by pathways involving phenylalanine metabolism, hormone metabolism and signaling under stress conditions. Furthermore, we found that the concentration of salicylic acid and two precursors, shikimic acid and phenylalanine, showed rapid negative accumulation after all three stresses. The expression levels of some key genes involved in salicylic acid metabolism and signal transduction were differentially expressed under stress conditions. This study extends our understanding of the mechanism of primary root responses to abiotic stress tolerance in maize.

**Supplementary Information:**

The online version contains supplementary material available at 10.1186/s12870-022-03805-4.

## Background

The negative impacts of abiotic stresses on plant growth and crop productivity are the main cause of extensive agricultural losses worldwide. Different abiotic stresses cause varying extents of damage to plant growth, and abiotic stresses in nature are often not isolated. Combinations of two or more stresses, such as drought and salinity, heat and salinity, and drought and extreme temperature or high light intensity, are common in many agricultural regions around the world. Climate prediction models indicate a gradual increase in ambient temperature and an enhancement in the frequency and amplitude of heat stress [1–3], and these increases are expected to continue in the future [4]. High temperatures will often be accompanied by other meteorological disasters, such as extended drought, and this combination of water deficit and heat stress will have profound impacts on crops by affecting plant growth and water use [5, 6]. For example, a lack of water induces stomatal closure, which reduces transpiration fluxes [7] and thus causes an increase in leaf temperature [8], which potentially enhances plant susceptibility to higher air temperature. In addition, the increase in leaf temperature can also increase the loss of plant water by transpiration [9] and decrease root growth [10], thus increasing the susceptibility of plants to water shortages.

In addition to physically supporting the plant, the root system also plays an important role in nutrient and water absorption and has a close relationship with the stress response. In coping with environmental stress, plants constantly optimize their unique root systems to satisfy their requirements for nutrients and water, and the sensitivity and tolerance to stresses are highly correlated with root development and plasticity to stresses [11]. The primary root is the first organ to appear at germination and the first to sense stress signals [12]. Under the conditions of drought stress, overall lateral root initiation and elongation were reduced, while the primary root was elongated to reach deeper water sources in the soil and to establish seedlings before shoot emergence [13, 14]. Studies have shown that it is unscientific to extrapolate the response to a combined stress directly from the response to each of the stresses applied individually, as the response of plants to a combination of different abiotic stresses is unique [15, 16]. Therefore, it is of great importance to understand the response mechanism of the root system to these complex stresses to improve the tolerance of crops.

Metabonomics and transcriptomics have attracted much attention when deeply exploring various complex reaction mechanisms and regulatory systems in plants. For instance, several key regulators have been revealed as potential targets for improving the heat tolerance of maize based on an atlas of genome-wide transcriptomic responses to heat stress [17]. *ZmWRKY106*, a maize WRKY transcription factor involved in multiple abiotic stress response pathways that reacted positively to drought and heat stress, was identified on the basis of maize drought de novo transcriptome sequencing data [18]. The accumulation of sucrose, maltose and glucose was significant in *Arabidopsis* exposed to drought, heat or the combination of both [15]. Several key metabolites, including carbohydrates, amino acids, and lipids, were differentially accumulated in soybean leaves because of drought and heat stress [19]. The level of metabolites in plants, such as reactive oxygen species (ROS), will change under adverse conditions such as drought and high temperature [20]. Some antioxidants regulate stomatal closure, signal transduction, hormone signaling, reproductive growth, and stress-related gene expression [21, 22]. Moreover, the regulation of hormones also plays an important role in the plant response to abiotic stress. Salicylic acid (SA) can regulate antioxidant defense systems in plants, and a number of studies have shown that exogenous SA stimulates plant growth under abiotic stress [23, 24].

As maize is a major food crop, it is important to uncover the mechanism of maize tolerance to abiotic stress. In this study, the response mechanism of maize primary roots to drought, heat and the combined stress of drought and heat was explored by combining metabolomics and transcriptomics analyses. The results will provide new insights into the drought and heat tolerance of maize.

## Results

### Primary root phenotype of the maize inbred line B73 under abiotic stress

**Fig. 1:**
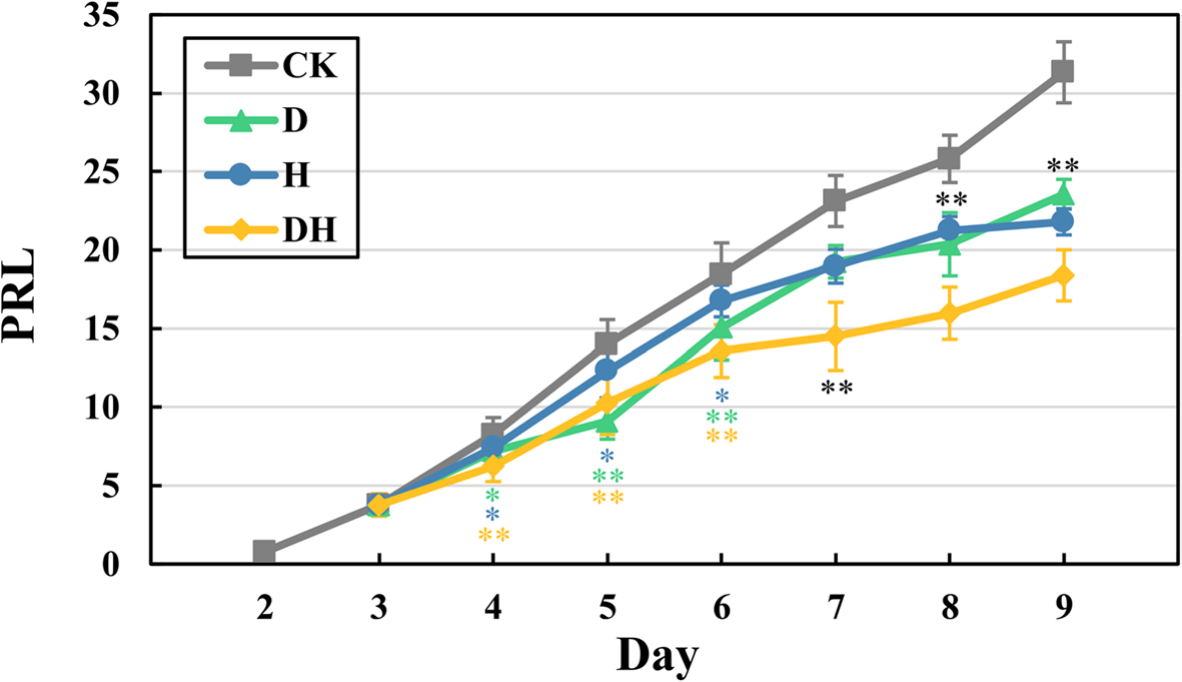
Dynamic change of the primary root lengths under control (CK), drought (D), heat (H) and combined stress (DH) during 9 days after germination. Significant differences in the average lengths at each time point were calculated using the *t*-test. Different colors of “*” and “**” indicated a significant difference (*P* < 0.05) and a highly significant difference (*P* < 0.01), respectively. Begin with day 7, compared with control, the stressed plants showed a highly significant difference, which were indicated by black “**” (*P* < 0.01)

To explore the effects of drought (D), heat (H) and combined stress (DH) on the growth of the primary root, the primary root length (PRL) of the maize inbred line B73 was evaluated in a time course experiment (Fig. 1). Three-day-old seedlings were subjected to drought stress treatments (PEG8000: − 0.8 MPa), heat stress treatments (day: 40 °C, night: 35 °C) and a combination of drought and heat stress. After one day of stress treatment, the PRL under drought and heat stress decreased significantly (*P* < 0.05) compared with the control plants, while the PRL of the combined stress treatments was extremely significantly reduced (*P* < 0.01). The PRL also reached an extremely significant level on Day 5 and Day 6 under drought conditions. On Day 7, the PRLs were significantly reduced under all stresses. On Day 9, the PRLs decreased significantly by 24.83%, 30.45% and 41.33% under water deficiency, heat stress and combined stress, respectively.

### Metabolic changes in the primary root in response to abiotic stress

**Fig. 2:**
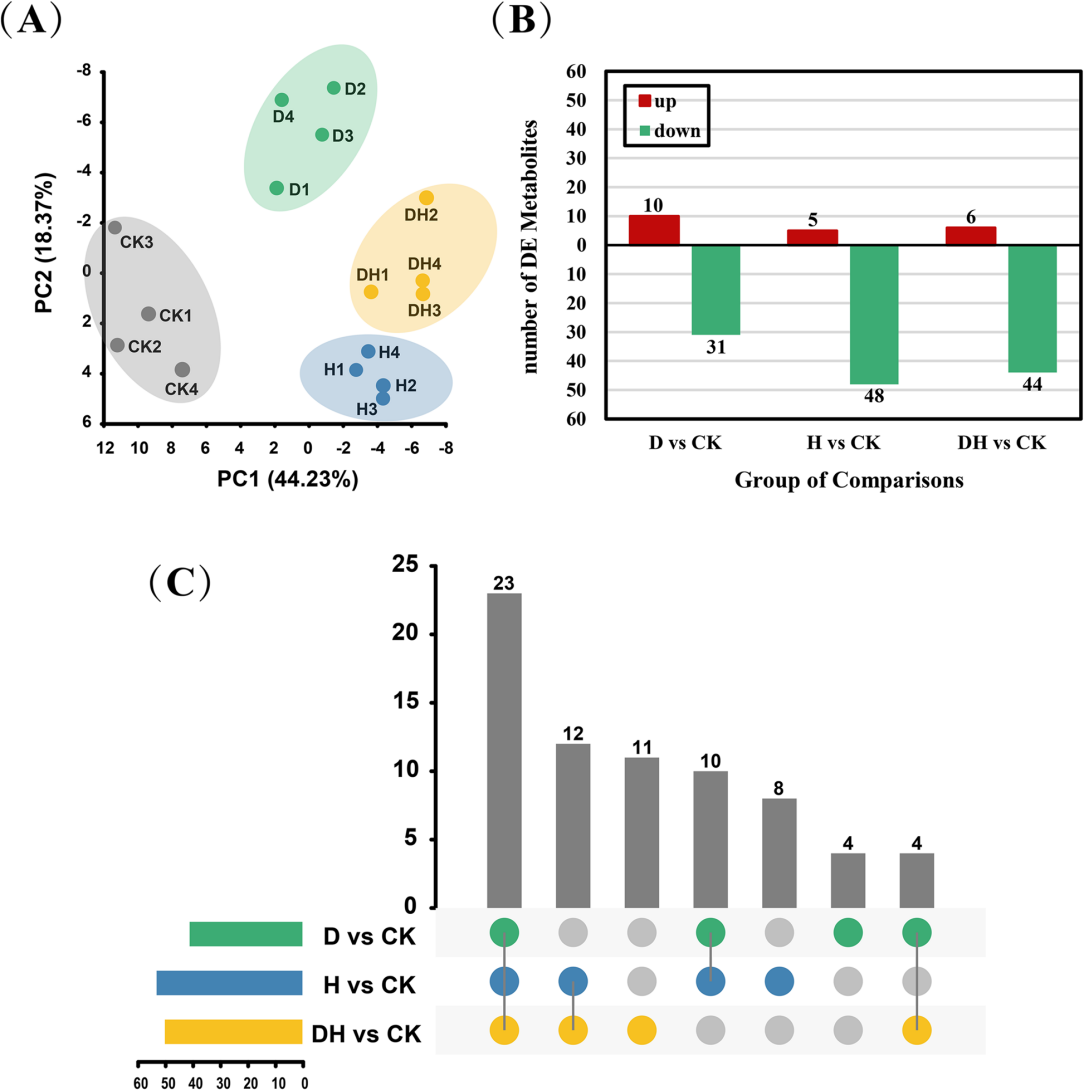
Correlation of root metabolism maps and DEMs under control and different stress treatments. **A** PCA analysis of biological repetitions after control, drought, heat and combined stress treatments. **B** The number of DEMs in the primary roots subjected water deficit, heat and combined stress compared with the control. Red indicated positive accumulation of DEMs and green showed the contents of DEMs decreased. **C** The number of DEMs and the overlap relationship among the comparison groups. The bar chart represented the number of DEMs contained simultaneously in the colored comparison group

To investigate the changes in metabolites in the primary roots subjected to abiotic stresses, we collected primary roots (treated with drought, heat and combined stress for 24 h) for metabolomic analysis, and 86 metabolites were obtained by GC–MS (gas chromatography—mass spectrometry). The annotated metabolites were divided into nine categories: amino acids, sugars, nucleotides, organic acids, fatty acids, phosphoric acids, amines, polyols, and others (Table S1). PCA was carried out on these 86 metabolites, and the first two principal components (PC1 and PC2) explained 44.23% and 18.37% of the variation, respectively (Fig. 2A). PCA showed a high correlation among the four biological replicates of each treatment, and roots subjected to different stresses could be clearly distinguished into different groups. Most of the metabolites showed a decreasing trend based on the standard *p* value ≤ 0.05 and VIP ≥ 1. We found that the contents of 10 metabolites were increased and 31 metabolites were decreased after drought stress, while the amounts of 5 and 48 metabolites increased and decreased in roots suffering from heat stress, respectively (Fig. 2B). The combination of drought and heat stress caused up- and downaccumulation of 6 and 44 metabolites, respectively. By cross-comparison of the differentially expressed metabolites (DEMs), we found that 23 metabolites responded to all three stresses (Fig. 2C). In addition, 4, 8 and 11 metabolites only responded to drought, heat and combined stress, respectively.

**Fig. 3:**
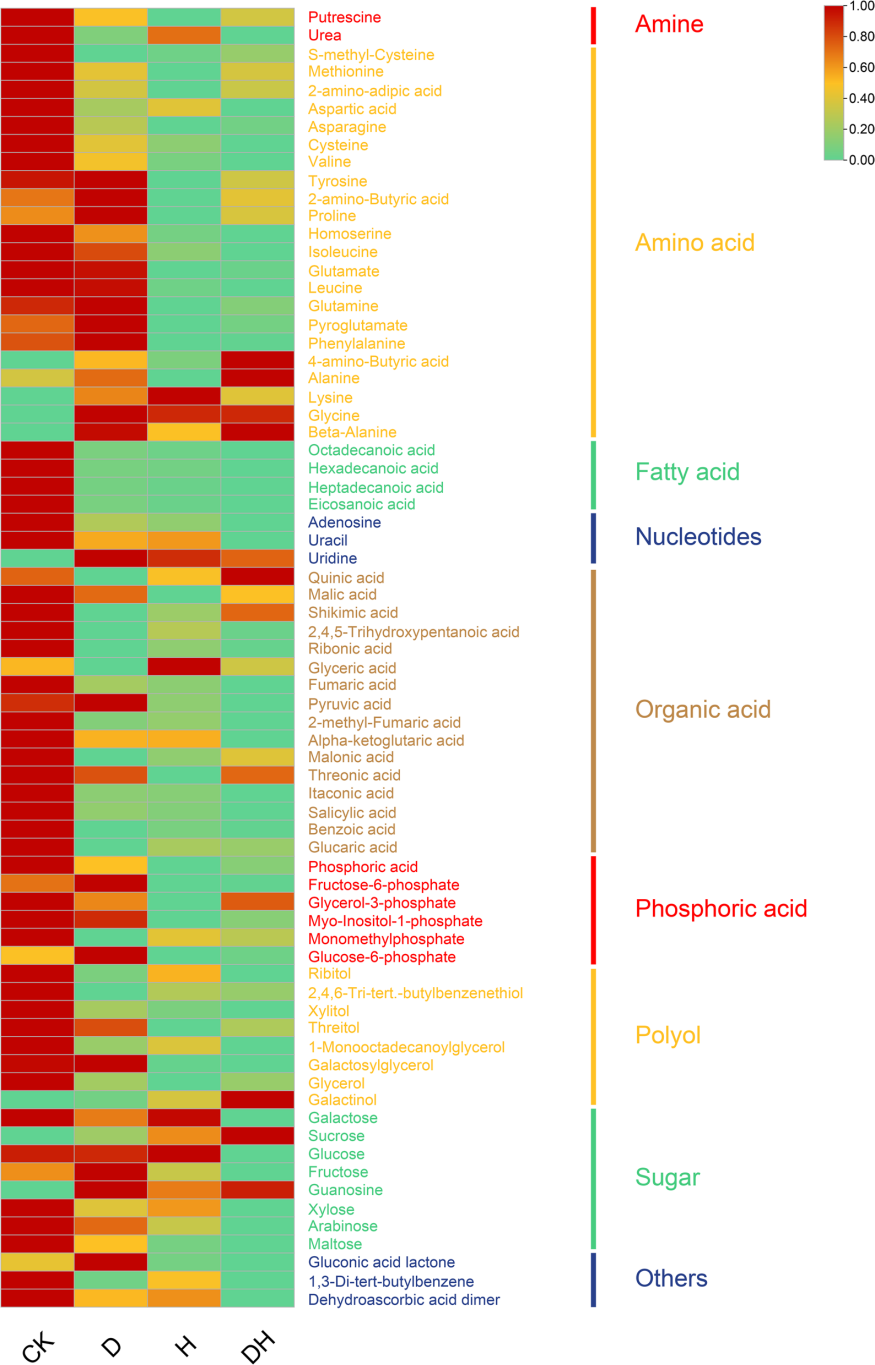
The content of DEMs in the primary roots with control, drought, heat and combined stress treatments. Cluster analysis of metabolites in the primary roots under different circumstances. The log_10_(Metabolite Content) of each metabolite was calculated to normalized the metabolite content. Red indicated metabolite with high content. The map was generated using the heatmap function of TBTools (Toolbox for Biologists v1.09876, http://www.tbtools.org/)

A total of 72 DEMs were detected, and they were divided into 9 categories, among which amino acids and organic acids were the most abundant. Most of the DEMs were downregulated under stress (Fig. 3). The contents of s-methyl-cysteine, methionine, 2-amino-adipic acid, aspartic acid, asparagine, cysteine, and valine decreased under the three kinds of stress, while the contents of lysine, glycine and beta-alanine increased under all the stresses. Tyrosine, 2-amino-butyric acid, proline, homoserine, isoleucine, glutamate, leucine, glutamine, pyroglutamate, and phenylalanine were downregulated under H and DH. In addition, 4-amino-butyric acid and alanine were upregulated under D and DH. In organic acids, 13 metabolites showed a decreasing trend, including malic acid, shikimic acid, 2,4,5-trihydroxypentanoic acid, ribonic acid, fumaric acid, 2-methyl-fumaric acid, alpha-ketoglutaric acid, malonic acid, threonic acid, itaconic acid, salicylic acid, benzoic acid, and glucaric acid, while only pyruvic acid, glyceric acid and quinic acid increased under D, H and DH, respectively. Among them, salicylic acid is an important plant hormone, and the response of SA to compound stress was greater than that to single stresses. The contents of two kinds of amines (putrescine and urea) and four fatty acids (octadecanoic acid, hexadecenoic acid, heptadecanoic acid, and eicosanoic acid) accumulated less under all three conditions. Among the three nucleotides, the contents of adenosine and uracil showed a negative trend, while uridine showed the opposite trend. For polyol, galactinol only accumulated under DH, both threitol and galactosylglycerol decreased under H and DH, and the contents of the other five metabolites decreased after all stress treatments. Among the 8 kinds of sugar, the contents of galactose and glucose decreased greatly under DH. Xylose, arabinose and maltose were downregulated under stress, especially under DH. Sucrose and guanosine were upregulated under all stresses. Fructose was upregulated under D but downregulated under H and DH. In addition, gluconic acid lactone followed the same trend as fructose. The contents of 1,3-di-tert-butylbenzene and dehydroascorbic acid dimer decreased after treatments.

### The key KEGG pathways of DEMs

**Fig. 4:**
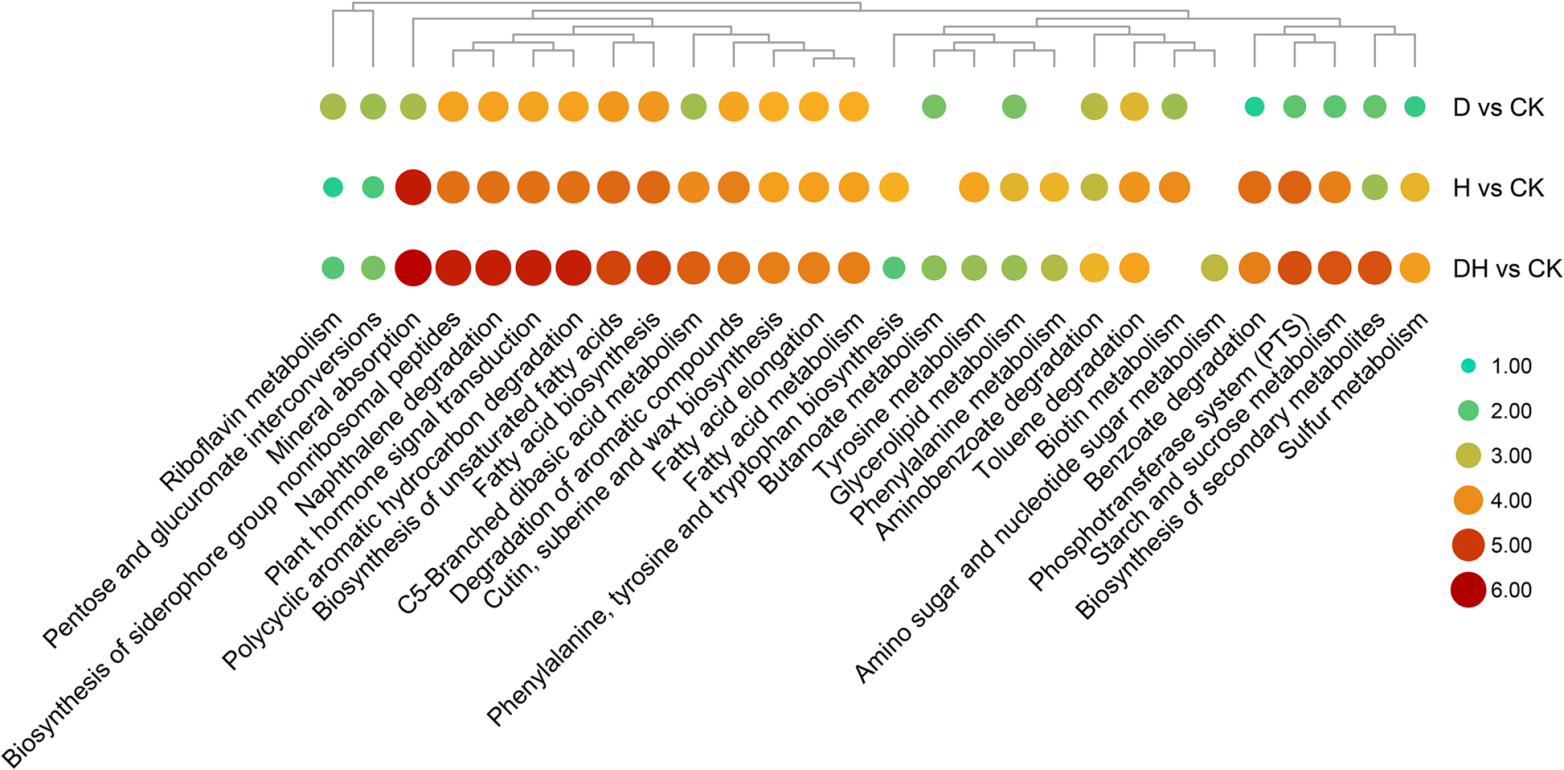
Enrichment of KEGG pathway of DEMs among comparison groups. According to the corrected *P*-value, the first 20 significant pathways of each group were selected and displayed in the figure. The -log_10_(*P*-value) of each comparison group in the selected pathway was calculated for the heat map. Red and large circular indicated that this pathway is more significant in the comparison between the two groups. The map was generated using the heatmap function of TBTools (Toolbox for Biologists v1.09876, http://www.tbtools.org/)

KEGG pathway analysis was performed to identify the main regulatory pathways in the primary roots under different environmental conditions. The top 20 significant pathways (*P* values < 0.05) in groups D_*vs*_CK, H_*vs*_CK and DH_*vs*_CK were selected for visualization (Fig. 4). The results revealed that the DEMs in the three groups were mostly involved in 85 metabolic pathways (Table S2). A total of 32 pathways were significantly enriched under all three stresses, including plant hormone signal transduction, biosynthesis of secondary metabolites, benzoate degradation, aminobenzoate degradation, and pentose and glucuronate interconversions. Phenylalanine metabolism, phenylalanine, tyrosine and tryptophan biosynthesis, and tyrosine metabolism were significantly enriched only between groups H_*vs*_CK and DH_*vs*_CK. Butanoate metabolism was significantly enriched in groups D_*vs*_CK and DH_*vs*_CK. In addition, amino sugar and nucleotide sugar metabolism was significantly enriched only in group DH_*vs*_CK.

### Global transcriptome analysis of the primary root of the maize inbred line B73 under abiotic stress

Transcriptome analysis was performed on the primary roots of B73 treated with drought, heat stress and a combination of both to investigate the gene expression profile. A total of 40.76–50.25 million clean reads were obtained for each of the 8 samples (Table S3). Compared with the maize reference genome (B73 RefGen_v3), 41,934 genes were identified and expressed at different levels in different tissues. A gene was considered to be active when the FPKM was greater than 1 in at least one sample. We found that a total of 26,644 genes were in an active state (Table S4).

**Fig. 5:**
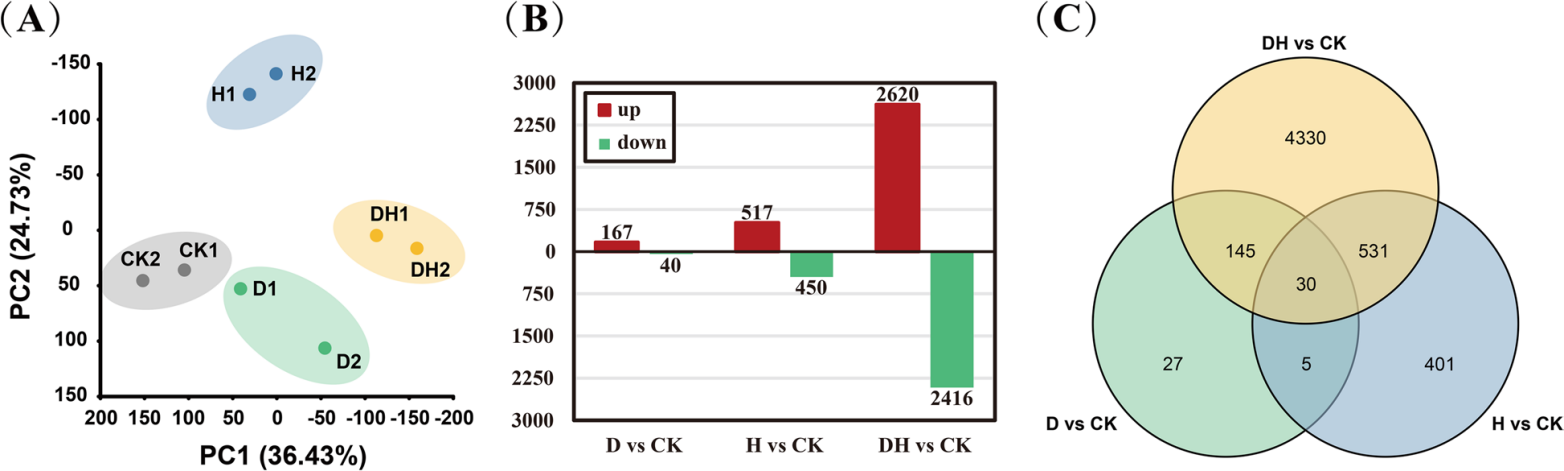
Global transcriptome analysis and the number of DEGs in three groups. **A** PCA analysis of biological repetitions after control, drought, heat and combined stress treatments. **B** The number of DEGs in the primary roots subjected water deficit, heat and combined stress compared with control plants. Genes up-regulated were denoted in red and down-regulated in green. **C** The number of DEGs and the overlap relationship among the comparison groups. Overlapping portions of the graph represented the number of DEGs in common among the comparison groups

We performed principal component analysis (PCA) on all active genes to assess the relationship of transcriptome samples. A total of eight samples from two biological replicates were used for drought, heat, combined stress and the control. Gene expression in the primary root was highly correlated between the two biological replicates for different treatments (Fig. 5A). PCA showed that there was a clear separation between the samples that received different stress treatments.

### Gene sets differentially expressed between abiotic stress treatments

There were biological differences in gene expression between the different treatments. Pairwise comparisons between control and stress treatment samples (D_*vs*_CK, H_*vs*_CK, and DH_*vs*_CK) were conducted to identify differentially expressed genes (DEGs). A total of 5,469 DEGs were detected (*p*-adjusted < 0.05), including 207,967 and 5,036 DEGs identified under drought, heat and a combination of both (Fig. 5B). We found 30 genes involved in all three types of stress. A total of 27,401 and 4,330 genes were only differentially expressed under drought, heat and the combined stress, respectively (Fig. 5C).

### Functional classification of DEGs among abiotic stress treatments

**Fig. 6:**
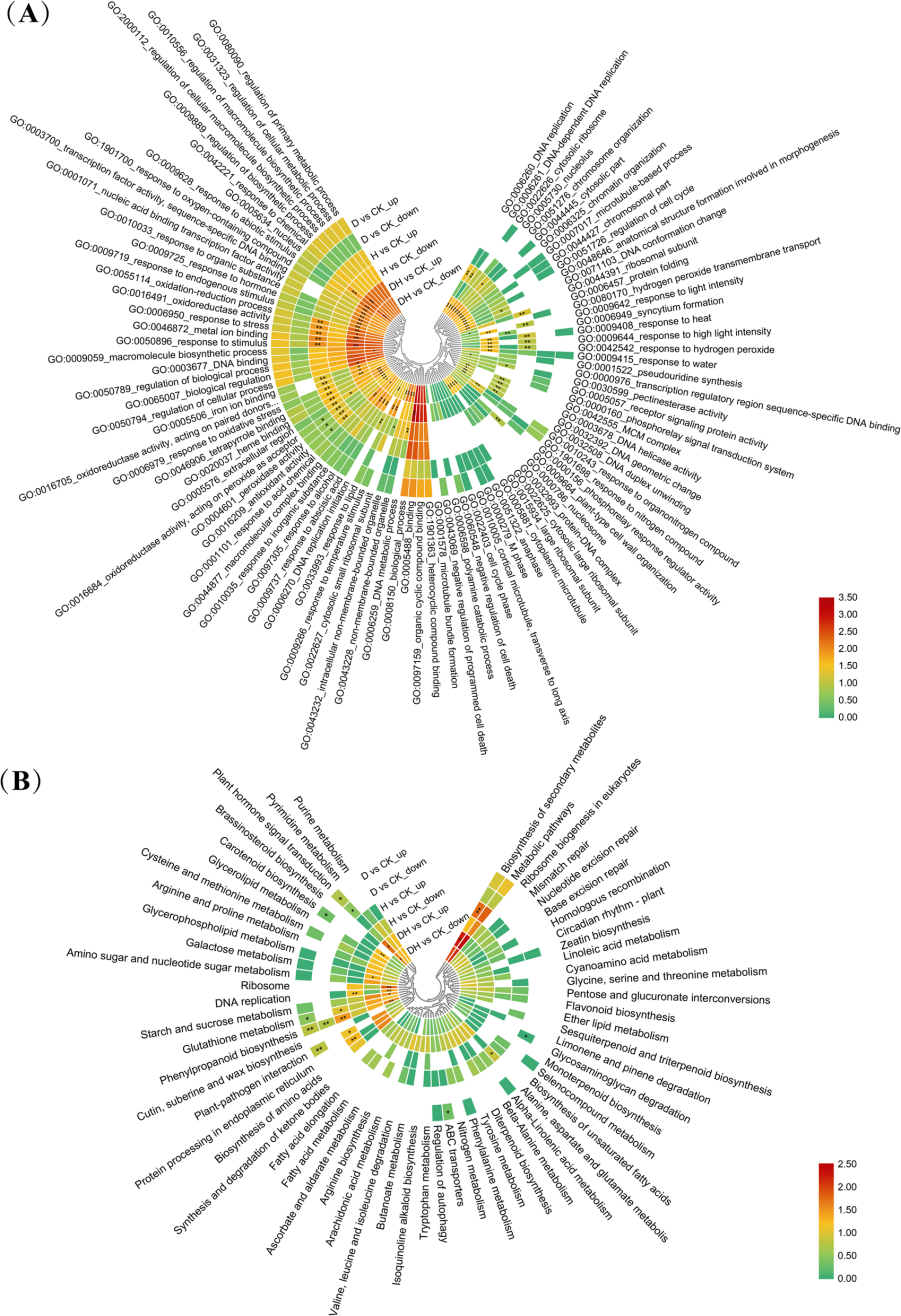
Functional enrichment of DEGs among each comparison group. **A** GO functional enrichment of DEGs among comparison groups. According to the corrected *P*-value, the first 20 GO terms in each comparison group were selected and shown in the figure. The heat map was drawn using calculate the log_10_(Gene Number) of each comparison group in the selected GO terms. Red indicated that this module contains more genes. “*” (*P* < 0.05) denoted significant enrichment of DEGs in this term, “**” (*P* < 0.01) denoted highly significant enrichment, otherwise not significant. **B** Enrichment of KEGG pathway of DEGs among comparison groups. According to the corrected *P*-value, the first 20 KEGG pathways per comparison group were selected and displayed in the figure. Calculate the log_10_(Gene Number) of each comparison group in the selected pathways and draw heat map. Red indicated that this module contains more genes. “*” (*P* < 0.05) means this pathway was significantly enriched, “**” (*P* < 0.01) means highly significant enrichment, otherwise not significant. The map was generated using the heatmap function of TBTools (Toolbox for Biologists v1.09876, http://www.tbtools.org/)

Functional enrichment of DEGs under different stresses was carried out by GOseq (Table S5). The top 20 GO terms for each stress were selected for visualization based on *P* values (Fig. 6A). The DEGs under drought stress were significantly enriched in oxidoreductase activity, acting on peroxide as an acceptor (GO:0,016,684), peroxidase activity (GO:0,004,601) and antioxidant activity (GO:0,016,209). There were 34 GO terms significantly identified under heat stress. Among them, metal ion binding (GO:0,046,872), response to stimulus (GO:0,050,896), oxidoreductase activity (GO:0,016,491), response to stress (GO:0,006,950), oxidation–reduction process (GO:0,055,114), and nucleus (GO:0,005,634) included more than 50 DEGs. We found 66 significant terms in the combined stress, and 39 terms included over 50 DEGs. Biological process (GO:0,008,150) and response to stimulus (GO:0,050,896) were two major terms that contained 1,404 and 415 DEGs, respectively. Among these functions, terms such as regulation of cellular process (GO:0,050,794, 291 DEGs), response to stress (GO:0,006,950, 236 DEGs), oxidoreductase activity (GO:0,016,491, 192 DEGs), response to abiotic stimulus (GO:0,009,628, 183 DEGs), and response to hormone (GO:0,009,725, 98 DEGs) were closely related to abiotic stress. In addition, 37 terms were significantly enriched only under compound stress, such as biological process (GO:0,008,150), DNA binding (GO:0,003,677), response to chemical (GO:0,042,221), nonmembrane-bounded organelle (GO:0,043,228), intracellular nonmembrane-bounded organelle (GO:0,043,232), and transcription factor activity, sequence-specific DNA binding (GO:0,003,700). At the same time, we found that the expression of some antioxidant related genes in plants changed after stress (Table S6), such as glutathione transferase activity (GO:0,004,364), peroxidase activity (GO:0,004,601), and response to reactive oxygen species (GO:0,000,302).

To further explore the biological functions of DEGs, as well as the major signaling pathways and biochemical pathways involved in these genes, we used KEGG to conduct pathway-based analysis (Table S7, Fig. 6B). In contrast with the control group, a total of 14 pathways were significantly enriched under stress treatments. The “plant hormone signal transduction” and “carotenoid biosynthesis” pathways were both significantly associated with drought and combined stress. The “DNA replication” and “biosynthesis of secondary metabolites” pathways were significantly correlated with heat stress and combined stress. The pathway “phenylpropanoid biosynthesis” was significantly identified among all stresses, while “galactose metabolism”, “ribosome” and “starch and sucrose metabolism” were only observed in the combined stress.

### WGCNA module analysis

**Fig. 7:**
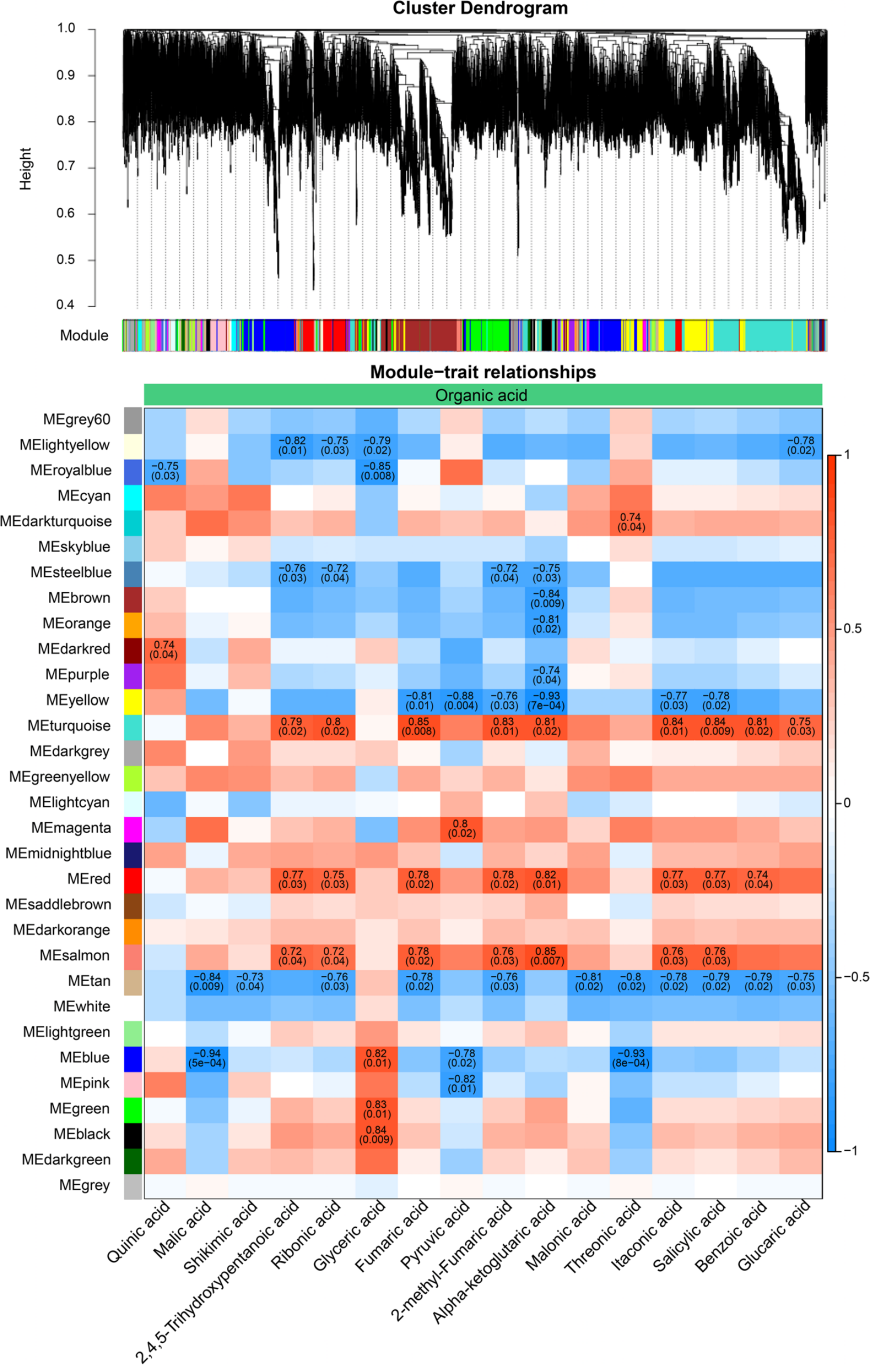
Weighted gene coexpression network analysis of the active genes (FPKM > 1). **A** Hierarchical clustering tree (cluster dendrogram) showing 31 modules of coexpressed genes according to their expression patterns. **B** Correlations between each module and organic acids. Pearson correlation coefficient and e-value of the module which had significant correlation with metabolites were shown in the figure. Red indicated positive correlation between modules and metabolites, blue indicated negative correlation

The active genes (FPKM > 1) were used for WGCNA, and 26,644 genes were divided into 31 modules according to their expression patterns (Fig. 7A). The correlation between the gene matrix of different modules and the DEMs was analyzed. We found that 19 modules were significantly associated with 72 DEMs, and each module was associated with 2–40 metabolites. Among them, 6 modules were significantly correlated with more than 20 metabolites, and genes in the modules “MEyellow”, “MEturquoise”, “MEtan”, “MEsalmon”, “MEred” and “MEblue” were involved in the regulation of 40, 37, 34, 33, 29 and 24 metabolites, respectively (Fig. 7B, Figures S1-S3). The module “MEyellow” was negatively correlated with 11 kinds of amino acids and 6 kinds of organic acids. Genes in the module “MEtan” were negatively correlated with 7 kinds of amino acids and 11 kinds of organic acids. Those in the module “MEturquoise” were positively correlated with 7 kinds of amino acids and 9 kinds of organic acids. Those in the module “MEblue” were negatively correlated with 12 amino acids. Genes in the module “MEsalmon” were positively correlated with 6 kinds of amino acids and 7 kinds of organic acids. Those in the module “MEred” were positively correlated with 8 organic acids. Among them, organic acids had the largest number of correlations with genes in these 6 modules, and we found that SA showed a significant positive correlation with genes in modules “MEturquoise” (0.84), “MEred” (0.77) and “MEsalmon” (0.76) and a negative correlation with genes in modules “MEyellow” (-0.78) and “MEtan” (-0.79).

**Fig. 8:**
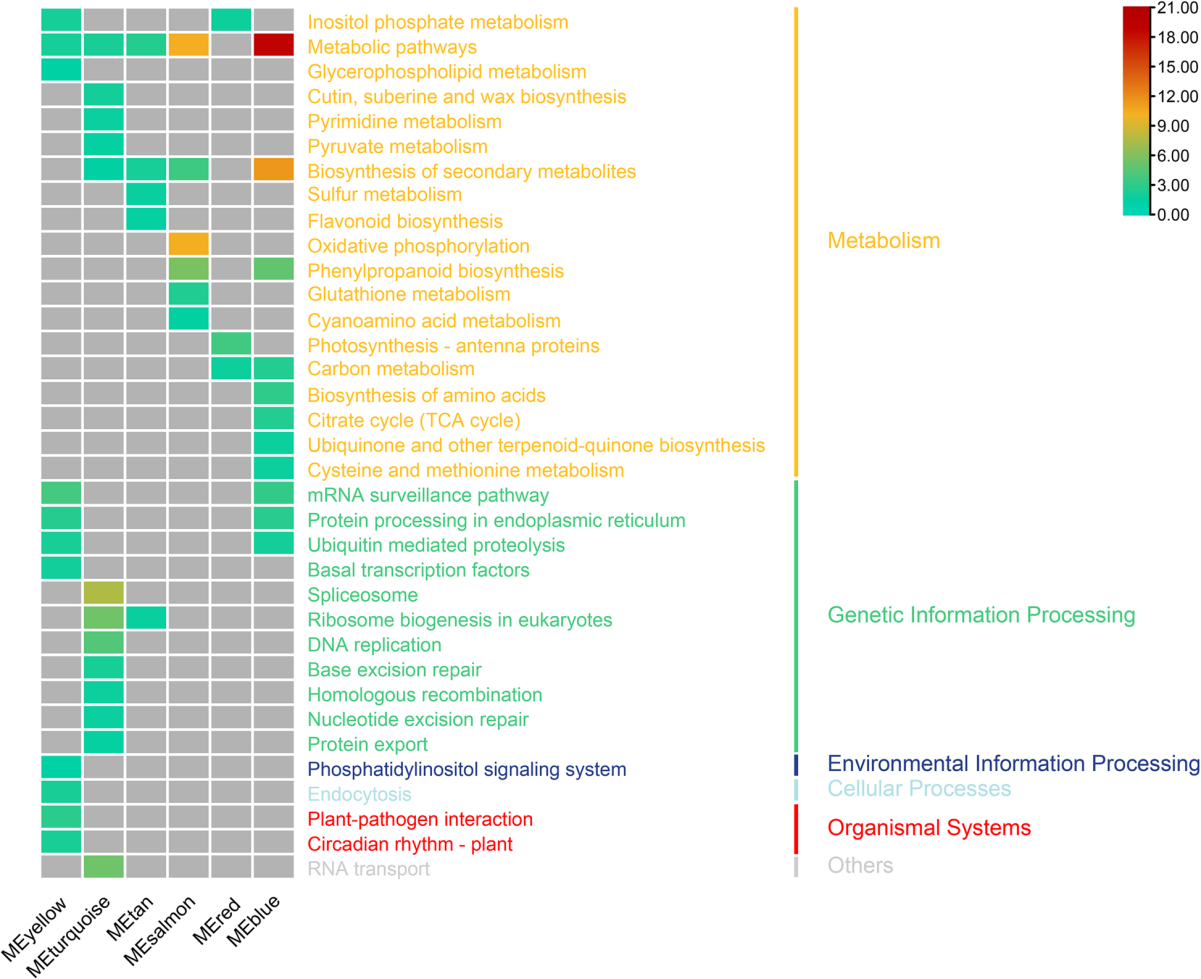
Enrichment of KEGG pathway of genes in the module “MEyellow”, “MEturquoise”, “MEtan”, “MEsalmon”, “MEred”, and “MEblue”. Calculate the -log_10_(Corrected *P*-Value) of significantly enriched pathways selected in each module and draw heat map (Ribosome was significantly enriched in the module “MEturquoise” but not shown in the figure because of the large magnitude). Closer to the red means more significant enrichment. The map was generated using the heatmap function of TBTools (Toolbox for Biologists v1.09876, http://www.tbtools.org/)

KEGG analysis was conducted to explore the biological functions of genes in the six modules (Fig. 8). Genes in the module “MEyellow” were associated with the largest number of metabolites and were significantly enriched in 11 metabolic pathways, such as glycerophospholipid metabolism, ubiquitin-mediated proteolysis, basal transcription factors, and plant–pathogen interactions. Those in the module “MEturquoise” were significantly enriched in 13 metabolic pathways, such as pyruvate metabolism, biosynthesis of secondary metabolites, nucleotide excision repair, and protein export, and those in the module “MEtan” were enriched in 5 metabolic pathways, including biosynthesis of secondary metabolites, flavonoid biosynthesis, and ribosome biogenesis, in eukaryotes. Genes in modules “MEsalmon” and “MEred” were only significantly enriched in the category metabolism. Genes in the module “MEsalmon” were enriched in 6 pathways, such as biosynthesis of secondary metabolites, oxidative phosphorylation, phenylpropanoid biosynthesis, and glutathione metabolism, and those in the module “MEred” were involved in inositol phosphate metabolism, photosynthesis-antenna proteins, and carbon metabolism. Genes in the module “MEblue” were mainly involved in 11 metabolic pathways, such as biosynthesis of secondary metabolites, citrate cycle (TCA cycle), ubiquinone and other terpenoid-quinone biosynthesis, and cysteine and methionine metabolism.

### Salicylic acid regulates root development under stress conditions

**Fig. 9:**
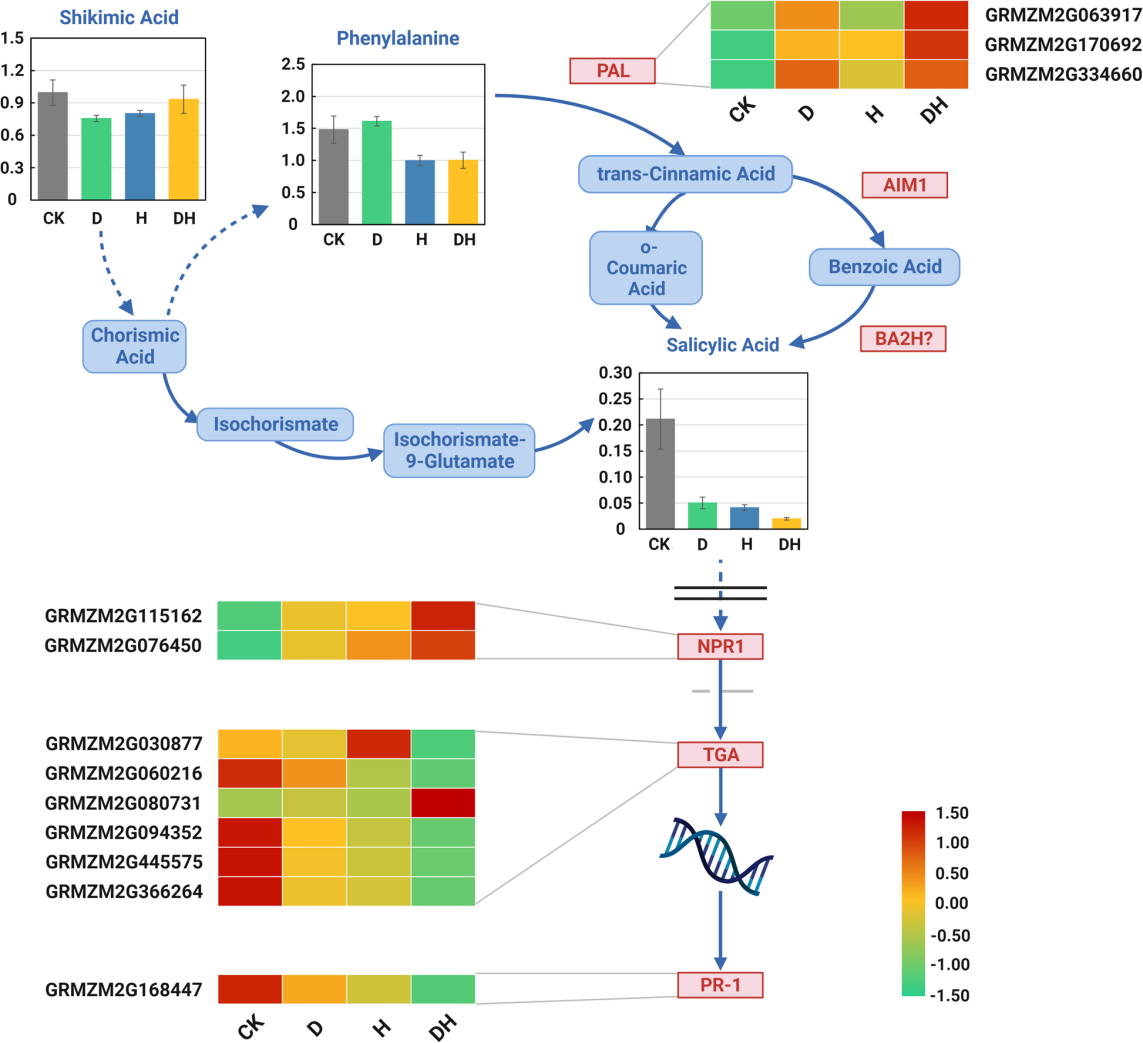
Salicylic acid biosynthesis and signal transduction pathway. The expression levels of DEGs were standardized, and the log_10_(Gene Expression) was calculated. Red represented genes with high expression. The black double line is the cell membrane and the grey dotted line is the nuclear membrane. The blue squares indicated that the metabolites were not measured. “?” indicated that an enzyme has not been confirmed. The pathway referred to the map00400, map00360, and map04075 in the KEGG database and we have obtained the permission from the Kanehisa laboratory

The functional annotation of DEGs and DEMs indicated that primary root development was mediated by pathways involving phenylalanine metabolism, hormone metabolism and signaling under stress conditions. We found that salicylic acid, an important plant hormone related to organic acids, showed a rapid reduction in accumulation after the three kinds of stresses. Shikimic acid and phenylalanine, which are involved in the synthesis of SA, were also reduced under stress conditions (Fig. 9). To further investigate the regulation of SA-related DEGs in primary root development under stress, we analyzed the expression patterns of DEGs related to salicylic acid biosynthesis and signal transduction. Phenylalanine produces trans-cinnamic acid in the presence of phenylalanine ammonia-lyase (PAL). We identified three PAL genes that were upregulated under stress, and they were classified into modules “MEbrown”, “MEyellow” and “MEred”. SA affected the expression of downstream genes after entering the cell membrane. Two NPR1 (Nonexpressor of Pathogenesis-Related 1) genes which were in the module “MEyellow”, were upregulated. Six TGA (transcription factor TGA) genes were identified. The expression patterns of GRMZM2G094352 (negatively correlated with the module “MEyellow”), and GRMZM2G445575, GRMZM2G366264, and GRMZM2G060216 (positively correlated with the module “MEturquoise”) were the same, and all of them were downregulated after treatment, especially under the combination treatment. Only one PR1 (pathogenesis-related protein 1) gene was identified, which played a role downstream of SA. PR1 was correlated with the module “MEturquoise”, and its expression was reduced under all stress conditions.

## Discussion

Plants experience a myriad of abiotic stresses in their life cycle, including water scarcity and high temperatures. These stresses can have a negative effect on crops, resulting in a significant reduction in yields. The root is an important organ used by plants to absorb water and nutrients and to sense and respond to stresses. Therefore, the molecular mechanism of plant roots in response to abiotic stresses has received increasing attention in recent years. Plants have evolved physiological, biochemical, and transcriptional processes to cope with various stresses in their surrounding environment. Drought and heat stress have been widely studied, while little is known about the effects of their combination on the primary roots of maize. In the present study, we investigated the single and combined effects of drought and heat stress on the growth of maize primary roots and conducted metabolomic and transcriptomic analyses to explore the molecular mechanisms.

### Effects of single and combined drought and heat stress on maize primary roots

Studies have shown that plant tolerance to adverse environmental conditions is highly correlated with root development and the regulation of stress response strategies [25]. The PRL of maize decreased significantly under drought stress, heat stress and compound stress, and the compound stress had a more serious negative impact on root length in this research. Vile et al. [26] dissected the effects of abiotic stresses on growth traits in Arabidopsis and found that plant growth was significantly reduced under both drought and heat stress, and their combination resulted in even more harmful effects. In fact, some effects occurred only under a single stress, indicating a certain degree of independence between the regulatory mechanisms of plant responses to drought and heat stress. For example, drought caused significant reductions in biomass, plant height and spike numbers, but there was no significant effect on these traits when barley was subjected to heat stress alone. In contrast, heat stress increased the number of aborted spikes and decreased kernel weight significantly, while drought did not [27]. Although there is a degree of overlap in the effects of a single stress on plants, each combination of two or more different stresses may also require a specific response [28].

### Metabolite changes and differential gene expression in maize primary roots under stress

Metabolomics and transcriptomics were performed on the primary roots of the maize inbred line B73 under a single stress of either drought or heat and their combination in the present research. We found that the content of most of the 9 kinds of differential metabolites decreased after stress. Especially for organic acids, the content of 16 metabolites showed a decreasing trend. Organic acids form a predominant part of root exudates and are intermediates of the tricarboxylic acid (TCA) cycle of cellular metabolism; many environmental stresses stimulate the biosynthesis of organic acids and their release from the roots [29]. Root organic acid exudation plays an important role in the plant response to climate change and the maintenance of crop yield. Organic acids balance excess ions in cells, are important in osmotic regulation, and can be excellent metal chelators in the soil [30]; organic acids help plants fix nitrogen. For example, malate influences primary root growth during Pi deficiency, acetic acid influences drought stress tolerance, and oxalate influences biotic stress tolerance [31–33]. The specific exudation of malic acid from the root tip could effectively improve the tolerance of plants to aluminum in soil [34]. It has been reported that the addition of sugar and organic acids could promote the absorption of nitrogen by plants, and the promoting effect of organic acids was more obvious [35]. Therefore, we speculate that the decrease in organic acid content is one of the important factors leading to the inhibition of maize primary roots under heat, drought, and combined stress.

Based on the enrichment of DEMs, we found that phenylalanine metabolism, phenylalanine, tyrosine and tryptophan biosynthesis, and tyrosine metabolism were only significantly enriched under H and DH, butanoate metabolism was only significantly enriched under D and DH, and amino sugar and nucleotide sugar metabolism was only significantly enriched under the combined stress. These results demonstrate that the relationship of plant responses to a single stress and compound stress are not simply additive. In addition, the enrichment of metabolic pathways under stress conditions, such as phenylalanine metabolism, pyruvate metabolism and the nitrogen metabolism pathway, also further demonstrated the importance of organic acids in the response of roots to stress.

Transcriptome analysis showed that, compared with the control group, 27, 401 and 4,330 genes were differentially expressed only under drought, heat and combined stress, respectively, and 30 DEGs were differentially expressed under all three types of stresses. This result suggested that the effect of compound stress on plant roots was much greater than that of a single stress. We found some functions related to abiotic stress by annotating and enriching the DEGs in the GO database. For instance, the GO terms “response to stress (GO:0,006,950)” and “response to heat (GO:0,009,408)” were upregulated only under heat stress and combined stress but were not significantly affected by water deficit. These genes may be more sensitive to high-temperature stimulation. Thirty-seven GO terms were only enriched under combined stress, such as biological process, DNA binding, response to chemical, nonmembrane-bound organelle, intracellular nonmembrane-bound organelle, transcription factor activity, and sequence-specific DNA binding. Single stresses did not have significant effects on these genes, but their expression patterns were unique under compound stress. To counter the harmful effects caused by drought, heat and salinity, plants change the levels of metabolites such as ROS [20]. Therefore, to eliminate and detoxify the negative effects of excessive ROS, plant cells develop a variety of effective defense systems, including enzymatic antioxidants such as superoxide dismutase (SOD), peroxidase (POX), catalase (CAT), ascorbate peroxidase (APX), glutathione reductase (GR) and nonenzymatic antioxidants such as glutathione, ascorbic acid, carotenoids and tocopherols [36]. In this study, genes related to oxidoreductase activity, acting on peroxide as an acceptor (GO:0,016,684), antioxidant activity (GO:0,016,209), glutathione transferase activity (GO:0,004,364), peroxidase activity (GO:0,004,601), and response to reactive oxygen species (GO:0,000,302) were differentially expressed after treatments.

### Analysis of gene pathways

Genes coordinate with each other to perform their biological functions in an organism. The KEGG database helped us mine the most important biochemical metabolic pathways and signal transduction pathways involving DEGs from gene and expression information and networks. We found that “glutathione metabolism” was significantly upregulated under drought and heat stress. Simultaneously, “plant hormone signal transduction” and “carotenoid biosynthesis” were only significantly correlated with drought and compound stress, and “carotenoid biosynthesis” showed positive responses to both kinds of stress environments. The nonenzymatic antioxidants glutathione and carotenoids [36] mitigate the toxicity of ROS, while plant hormones also play an important role in helping plants adapt to adverse environmental conditions. In addition, the DEGs related to “biosynthesis of secondary metabolites” in this study were significantly upregulated under heat stress and compound stress. Plant secondary metabolites are commonly referred to as compounds that have no fundamental role in sustaining plant life but are important for plants to adapt and defend themselves by interacting with the environment [37].

### Salicylic acid and abiotic stress in plants

By combining the coexpression modules with organic acids, we found that the modules we focused on were enriched in pyruvate metabolism, biosynthesis of secondary metabolites, phenylpropanoid biosynthesis, and glutathione metabolism. These pathways are closely related to the antioxidant defense system of plants. At the same time, we found that SA (organic acid) was also an important metabolite involved in the antioxidant defense mechanism of plants. As an endogenous plant hormone that regulates growth, it has significant effects on resistance to abiotic stress by affecting physiological and biochemical processes in plants [38]. Moreover, by regulating the antioxidant defense system, the application of SA was shown to alleviate adverse effects in drought-stressed *A. thaliana* [39] and *T. aestivum* [40] and heat-stressed *Z. mays* [41], *Poa pratensis* [42] and *Cucumis sativa* [43]. Exogenous SA stimulated root growth under abiotic stress [44, 45] and protected the root length of barley from the adverse effects of the combination of heat, drought and salt stress [38]. We investigated the metabolite content under three abiotic stresses (drought, heat and combined stress) and found that SA significantly decreased after stress treatments. The influence of combined stress was greater than that of a single stress. SA is synthesized from phenylalanine and benzoate as direct precursors [46, 47]. We detected that the content of phenylalanine significantly decreased under heat and combined stress but did not significantly change under drought. The expression levels of the three PALs were increased, but downstream trans-cinnamic acid was not detected. The underlying mechanism remains to be further studied. In addition, TGA transcription factors are involved in the regulation of plant root growth, stress response, flowering regulation and other physiological metabolic processes. Stotz et al. [48] showed that TGA2 transcription factors participated in the oxidative stress pathway and the REDOX regulation pathway of ROS induced by environmental stress in *Arabidopsis thaliana*, restoring plant growth and development to avoid the impact of external adverse conditions on plants. In tobacco, glyoxalase (GLO) promoted the interaction between TGA and NPR1 and recognized the specific sequence TGACGT to activate the expression of PR1 and to respond to the ROS pathway in plants [49]. In the present research, the expression of most TGA transcription factors decreased under stress, while the expression of NPR1s and GRMZM2G080731 increased. Studies have shown that SA in organisms inhibits the interaction between TGA and NPR1, weakens the expression of ROS deoxidization-related genes, inhibits the recovery of the ROS oxidative reduction state in organisms, and affects the growth and development of plants [50]. Therefore, the decrease in SA under stress may weaken the repression of NPR1s and some TGAs. Furthermore, it was found that coinoculation of *Azospirillum brasilense* and *Rhizobium tropici* may mitigate salinity stress in maize [51]. Under drought stress, the expression of PR1, PR2 and PR5 was directly upregulated in the lines overexpressing the transcription factor Di19, which improved the tolerance of Arabidopsis to drought [52]. Transgenic tobacco overexpressing SpMYB accumulated higher levels of pathogenesis-related gene transcripts than wild-type plants, such as PR1 and PR2, which enhanced salt and drought stress tolerance [53]. In the present research, we found that the PR induced by SA and NPR1 [54] was significantly downregulated under stress treatments. Although NPR1s downstream of SA were overexpressed, the activation of SA was required for its function [55, 56]. This may also be one of the important mechanisms of maize root response to drought, heat and combined stress at the seedling stage, while the specific regulatory mechanism needs to be further verified.

## Conclusions

In this study, maize seedlings were treated with control, drought, heat and a combination of both, and the results showed that the primary roots of maize plants were significantly reduced by 24.83%, 30.45% and 41.33%, respectively. A total of 72 differentially expressed metabolites detected in the primary root involved 85 metabolic pathways, such as plant hormone signal transduction, biosynthesis of secondary metabolites, and phenylalanine metabolism. Among them, the concentrations of salicylic acid, shikimic acid, and phenylalanine were reduced after stress. While a total of 207, 967, and 5,036 differentially expressed genes were identified under different stresses respectively, and these genes related to the regulation of pathways such as glutathione metabolism, plant hormone signal transduction, and carotenoid biosynthesis. The expression levels of some key genes involved in salicylic acid metabolism and signal transduction were differentially expressed under stress conditions. The decrease of salicylic acid may affect the expression of downstream TAG transcription factors and the binding of TAG to NPR1, which affects the expression of PR1, leading to the decreased tolerance of maize to abiotic stress. The results provide a theoretical basis for studying the response mechanism of maize taproots to abiotic tolerance.

## Methods

### Plant material, growth conditions, treatment, and measurement of primary root length

Phenotypic, transcriptomic, and metabonomic analyses were carried out using the maize inbred line B73. Seeds with uniform size and intact embryos were selected and sterilized with 10% H_2_O_2_ for 30 min. The seeds were rinsed several times with distilled water to remove H_2_O_2_ from the surface of the seeds. The sterilized seeds were soaked in saturated CaSO_4_ for 6 h to promote germination and then placed on moist filter paper at 28 °C and 80% relative humidity for 2 days. After 2 days, the germinated seeds were rolled up with brown germinating paper (Anchor Paper Company, St Paul, MN, USA) at 8 seeds per roll. The hydroponic experiment was conducted in an incubator at 28/22 °C (day/night) with a relative humidity of 60% and a light intensity of 400 μmol.m^−2^.s^−1^. On Day 3, Hoagland solution was used instead of distilled water. At the same time, PEG8000 solution (-0.8 MPa) was added to simulate the water deficit, and the incubator was set at 40 °C as the daytime temperature and 35 °C at night to simulate the heat environment or a mixture of the two treatments. The nutrient solution was replaced every 2 days. Four independent biological replicates were set for each treatment, and the root traits were determined in the paper-roll system [57]. On Day 4, 30 primary roots per treatment were collected for each biological replicate. Two and 4 independent biological replicates were analyzed for transcriptomics and metabolomics, respectively. All root samples were immediately frozen in liquid nitrogen and stored in a cryogenic refrigerator at -80 °C.

### Sample extraction and detection of the metabolome

One hundred milligrams of fresh root samples and 5 steel balls were placed in 5 mL centrifuge tubes, frozen in liquid nitrogen for 5 min and then transferred to a high flux grinding apparatus (SCIENTZ-48, Ningbo Scientz Biotechnology Co., LTD, China) for grinding (70 Hz 1 min). Then, 1,400 µL of methanol (precooled at -20 °C) and 60 µL of the internal standard Ribitol (0.2 mg/mL stock in methanol) were added successively and vortexed for 30 s each. Next, the tubes were placed in an ultrasound machine at room temperature for 30 min and 750 µL of chloroform (precooled at -20 °C) and 1,400 µL of deionized water (dH_2_O) (4 °C) were added before vortexing for 1 min. One milliliter of supernatant was transferred to a 1.5 mL centrifuge tube after centrifugation (14,000 rpm, 4 °C for 10 min) and then blow-dried by a vacuum concentrator. The solution was reacted for 2 h at 37 °C after vortexing for 30 s with 60 µL of methoxyamine pyridine solution. Finally, 60 µL of BSTFA reagent (containing 1% TMCS) was added and reacted at 37 °C for 90 min. After centrifugation for 10 min (12,000 rpm, 4 °C), the supernatant was transferred to the inspection bottle for GC–MS test detection. Gas chromatography was performed on an HP-5MS capillary column (5% phenyl/95% methylpolysiloxane 30 m × 250 μm i.d., 0.25 μm film thickness, Agilent J & W Scientific, Folsom, CA, USA) to separate the derivatives at a constant flow of 1 mL/min helium. One microliter of sample was injected in split mode in a 20:1 split ratio by the autosampler. The injection temperature was 280 °C, the interface was set to 150 °C, and the ion source was adjusted to 230 °C. The temperature-rise program was followed with an initial temperature of 60 °C for 2 min, a 10 °C/min increase up to 300 °C and a hold at 300 °C for 5 min. Mass spectrometry was determined by the full-scan method with a range from 35 to 750 (m/z).

### Sample extraction and detection of the transcriptome

Total RNA of maize primary roots was extracted with an RNeasy Plant Mini Kit (Qiagen, Shanghai, China). A total of 3 µg of RNA per sample was employed as the input material for RNA sample preparation. The sequencing library was established by the NEBNext® Ultra™ RNA Library Prep Kit for Illumina® (NEB, USA). Library fragments were purified using the AMPure XP system (Beckman Coulter, Beverly, USA) to preferentially single out cDNA fragments 150–200 bp in length. Size-selected adaptor-ligated cDNA was reacted with 3 µg of USER Enzyme (NEB, USA) at 37 °C for 15 min and then at 95 °C for 5 min, followed by PCR performed with Phusion High-Fidelity DNA polymerase, Universal PCR primers and Index (X) Primer. Finally, PCR products were purified by the AMPure XP system, and library quality was assessed on the Agilent Bioanalyzer 2100 system. After clustering the indexed-coded samples, the Illumina HiSeq platform was used for sequencing to generate 125 bp/150 bp paired-end reads.

### Metabonomic data analysis

Raw data were converted to netCDF format (xcms input format) by Agilent MSD ChemStation. The XCMS R package (v3.1.3) was used for peak identification, peak filtration, and peak alignment. The data matrix comprising the mass to charge ratio (m/z), retention time and intensity was used to annotate the metabolites combined with the AMDIS program based on the National Institute of Standards and Technology (NIST) commercial database and Wiley Registry metabonomic database. Most of the substances were further confirmed by the standard, and the data were exported for subsequent analyses.

### Transcriptome data analysis

From the raw data, reads containing adapters, reads with a proportion of poly-N (N represents undetermined clip information) greater than 10% and low-quality reads were removed to obtain clean reads. All subsequent analyses were based on clean, high-quality data. TopHat v2.0.12 was used for genomic mapping analysis, and paired-end clean reads were directly aligned to the reference genome downloaded from the genome website. The read numbers mapped to each gene were counted using HTSeq v0.6.1, and then the gene expression level was calculated based on the length of each gene to obtain FPKM (expected number of fragments per kilobase of transcript sequence per million base sequenced). RNA-Seq data have been deposited in the NCBI sequencing read archive (SRA) under accession number PRJNA645641.

The Pearson correlation coefficient between samples was calculated, and if the correlation was greater than 0.8, biological repetitions were considered effective. Differential expression analysis was performed by the DESeq R package (1.18.0). Benjamini and Hochberg’s methods were used to adjust the resulting *P* value to control the error discovery rate. Genes with an adjusted *P* value below 0.05 were considered differentially expressed. If the log2Foldchange > 0, the differentially expressed gene (DEG) is considered to be upregulated; the opposite suggests that the DEG is downregulated. The GOseq R package was used for Gene Ontology (GO) functional enrichment analysis of the DEGs, while the pathway-based KEGG (Kyoto Encyclopedia of Genes and Genomes, https://www.kegg.jp/) [58, 59] enrichment analysis was performed by KOBAS (http://kobas.cbi.pku.edu.cn/kobas3). When the *P* value after correction was less than 0.05, the DEGs were considered to be significantly enriched. The WGCNA R package was used to perform weighted gene coexpression network analysis (WGCNA) of the DEGs.

### Research involving plants

Experimental research and the material comply with relevant institutional, national, and international guidelines and legislation. And we have permission to collect seeds used in current study.

## Supplementary Information

**Additional file 1.**

**Additional file 2.**

## Data Availability

The data sets supporting the results of this article are included within the article (and its supplementary files). Raw reads of all samples have been deposited into the NCBI Sequence Read Archive (SRA, http:www.ncbi.nlm.nih.gov/sra/) under accession number PRJNA645641. All data generated or analysed during this study are included in this published article [and its supplementary information files].

## Abbreviations

ROS: Reactive oxygen species
SA: Salicylic acid
FPKM: Expected number of fragments per kilobase of transcript sequence per million base sequenced
SRA: Sequencing read archive
DEG: Differentially expressed gene
GO: : Gene ontology
KEGG: Kyoto Encyclopedia of Genes and Genomes
WGCNA: Weighted Gene Co-Expression Network Analysis
CK: Control
D: Drought
H: Heat
DH: The combination of drought and heat stress
PRL: The primary root length
PCA: Principal component analysis
GC–MS: Gas chromatography—mass spectrometry
DEM: Differentially expressed metabolites
PAL: Phenylalanine ammonia-lyase
NPR1: Nonexpressor of Pathogenesis-Related 1
PR1: Pathogenesis-related protein 1
SOD: Superoxide dismutase
POX: Peroxidase
CAT: Catalase
APX: Ascorbate peroxidase
GR: Glutathione reductase
GLO: Glyoxalase
