# Supplementary material for: Primary root response to combined drought and heat stress is regulated via salicylic acid metabolism in maize
Source: BMC Plant Biol. 2022 Aug 30;22:417. doi: 10.1186/s12870-022-03805-4 (PMC9425997; doi:10.1186/s12870-022-03805-4)
Supplement: Supplementary file 1 — Additional file 1. [file 12870_2022_3805_MOESM1_ESM.pdf]

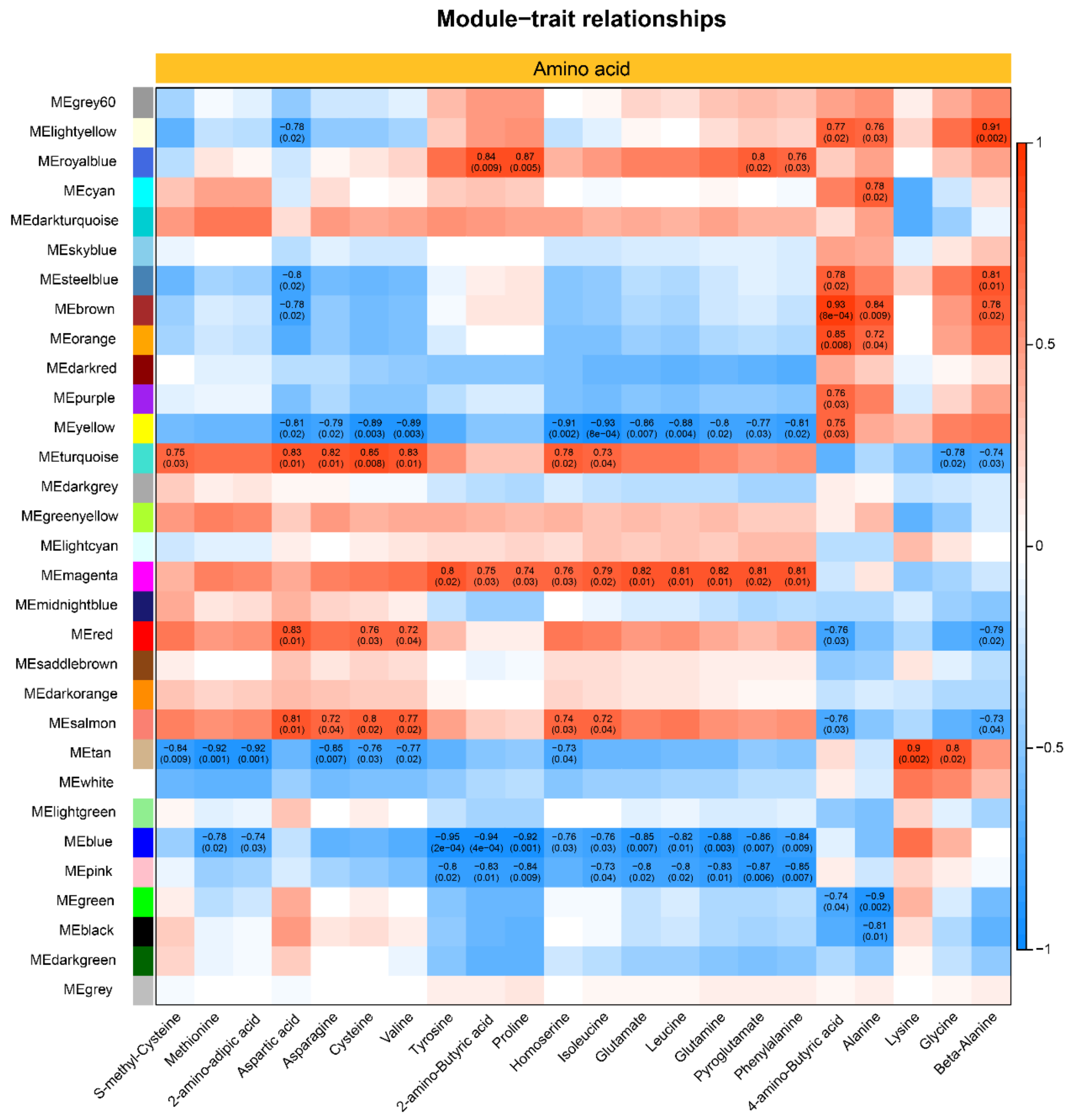

Fig.S1 Correlations between each module and amino acids. Pearson correlation coefficient and e-value of the module which had significant correlation with metabolites were shown in the figure. Red indicated positive correlation between modules and metabolites, blue indicated negative correlation.

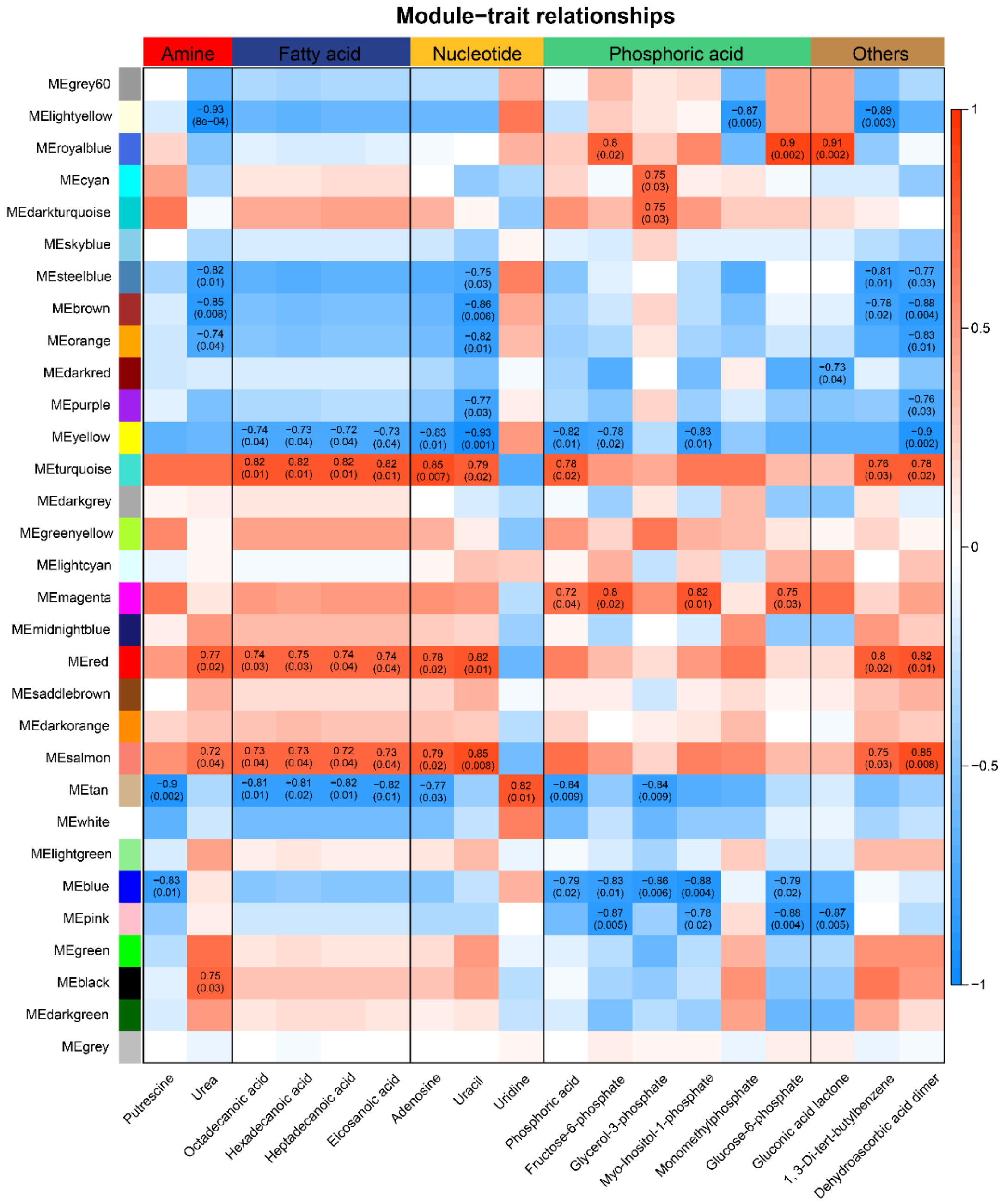

Fig.S2 Correlations between each module and amine, fatty acids, nucleotide, phosphoric acids, and others. Pearson correlation coefficient and e-value of the module which had significant correlation with metabolites were shown in the figure. Red indicated positive correlation between modules and metabolites, blue indicated negative correlation.

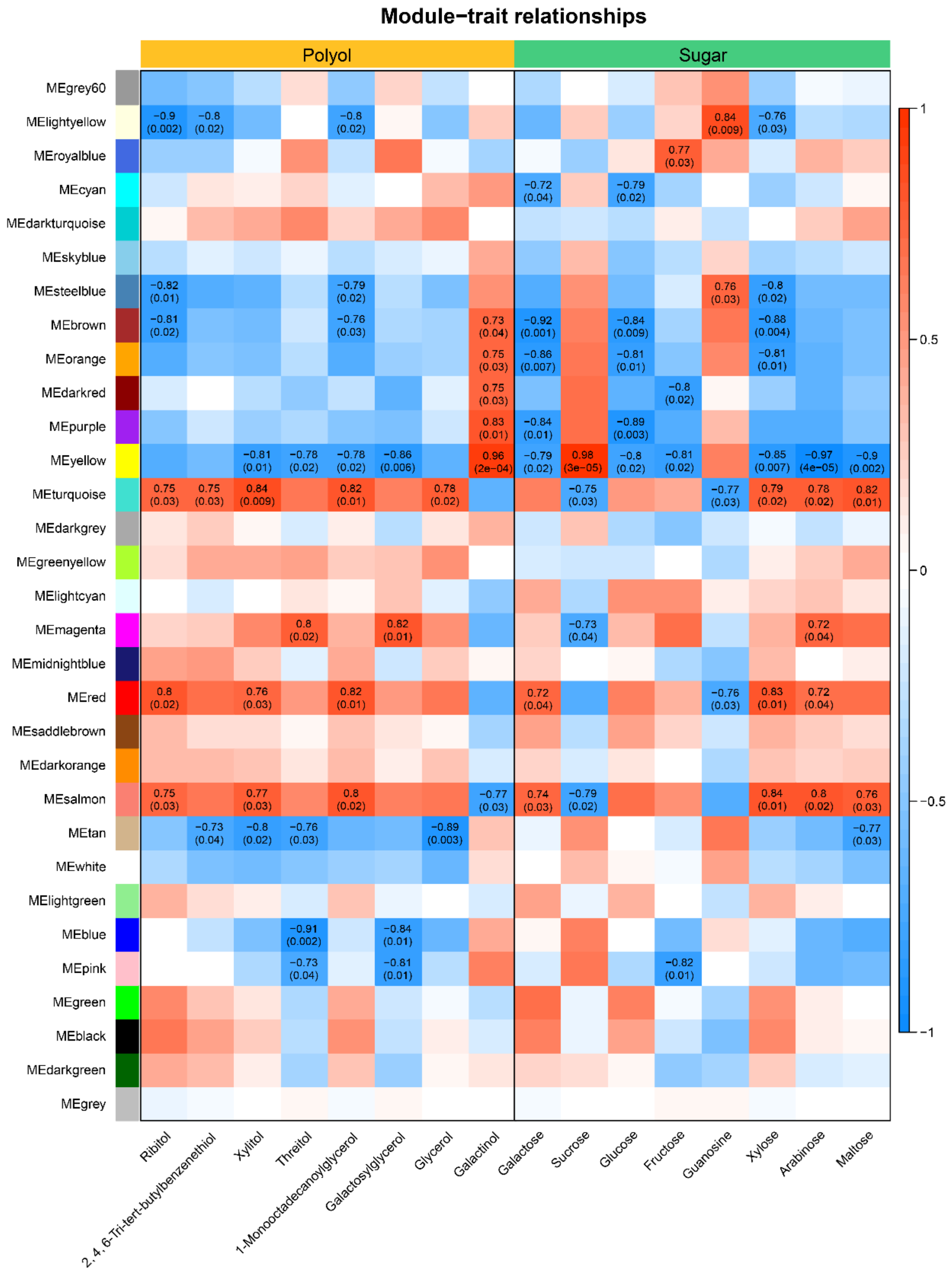

Fig.S3 Correlations between each module and polyols and sugars. Pearson correlation coefficient and e-value of the module which had significant correlation with metabolites were shown in the figure. Red indicated positive correlation between modules and metabolites, blue indicated negative correlation.
